# Structural Studies of Monounsaturated and ω-3 Polyunsaturated Free Fatty Acids in Solution with the Combined Use οf NMR and DFT Calculations—Comparison with the Liquid State

**DOI:** 10.3390/molecules28166144

**Published:** 2023-08-20

**Authors:** Themistoklis Venianakis, Michael G. Siskos, George Papamokos, Ioannis P. Gerothanassis

**Affiliations:** Section of Organic Chemistry and Biochemistry, Department of Chemistry, University of Ioannina, 45110 Ioannina, Greece; vethemis@gmail.com (T.V.); msiskos@uoi.gr (M.G.S.)

**Keywords:** 1D ^1^H NOE, ^1^H NMR chemical shift, ALA, EPA, DHA, DFT

## Abstract

Molecular structures, in chloroform and DMSO solution, of the free fatty acids (FFAs) caproleic acid, oleic acid, α-linolenic acid, eicosapentanoic acid (EPA) and docosahexaenoic acid (DHA) are reported with the combined use of NMR and DFT calculations. Variable temperature and concentration chemical shifts of the COOH protons, transient 1D NOE experiments and DFT calculations demonstrate the major contribution of low molecular weight aggregates of dimerized fatty acids through intermolecular hydrogen bond interactions of the carboxylic groups, with parallel and antiparallel interdigitated structures even at the low concentration of 20 mM in CDCl_3_. For the dimeric DHA, a structural model of an intermolecular hydrogen bond through carboxylic groups and an intermolecular hydrogen bond between the carboxylic group of one molecule and the ω-3 double bond of a second molecule is shown to play a role. In DMSO-d_6_ solution, NMR and DFT studies show that the carboxylic groups form strong intermolecular hydrogen bond interactions with a single discrete solvation molecule of DMSO. These solvation species form parallel and antiparallel interdigitated structures of low molecular weight, as in chloroform solution. This structural motif, therefore, is an intrinsic property of the FFAs, which is not strongly affected by the length and degree of unsaturation of the chain and the hydrogen bond ability of the solvent.

## 1. Introduction

Free fatty acids (FFAs) are carboxylic acids with short, medium, and long saturated or unsaturated aliphatic chains with 4 to 28 carbon atoms, which are stored as triacylglycerol in adipose tissue. Saturated, mono- and polyunsaturated FFAs, in the form of glycerolipids and phospholipids, are the major lipid components of cell membranes [1,2,3,4]. Fatty acids play essential roles in maintaining the correct membrane fluidity and environment for membrane protein function, and have essential roles in the regulation of energy, metabolism, inflammation, neurological and cardiovascular diseases [3,4,5,6,7,8,9]. Omega-3 FFAs are polyunsaturated fatty acids (PUFAs) which are characterized by the presence of a double bond featuring three carbon atoms from the terminal CH_3_ group. Three of the most important ω-3 PUFAs for human diet and physiology are α-linolenic acid ((9*Z*,12*Z*,15*Z*)-octadeca-9,12,15-trienoic acid, ALA), eicosapentaenoic acid ((5*Z*,8*Z*,11*Z*,14*Z*,17*Z*)-icosa-5,8,11,14,17-pentaenoic acid, EPA) and docosahexaenoic acid ((4*Z*,7*Z*,10*Z*,13*Z*,16*Z*,19*Z*)-docosa-4,7,10,13,16,19-hexaenoic acid, DHA). ALA is widely distributed in plants, while DHA and EPA are found in algae and fish [1,2,3,10,11].

The structural and conformational properties of unsaturated and ω-3 FFAs have been investigated with the use of ^1^H and ^13^C NMR spectroscopy [10,11,12,13,14], molecular dynamics and molecular mechanics [15,16,17]. NMR and computational studies have been reported of mono- and polyunsaturated FFAs bound to human and bovine serum albumin and in competition with various drugs [18,19]. Researchers have used a combination of various physicochemical techniques and molecular dynamics simulations to investigate membranes of 1-stearoyl(d_35_)-2-docosahexaenoyl-*sn*-glycero-3-phosphocholine and 1-stearoyl(d_35_)-2-docosapentaenoyl-*sn*-glycero-3-phosphocholine [20]. Law et al. [21] performed detailed DFT studies of a variety of conformations of ω-3 polyunsaturated free fatty acids. Translational motion, molecular conformation, and interdigitated hydrogen bonded aggregates in the liquid state of n-saturated and unsaturated free fatty acids were investigated with the use of ^13^C NMR spin-lattice relaxation times, self-diffusion coefficients and X-ray diffraction at various temperatures [22,23]. Raman spectroscopy and differential scanning calorimetry [24] and 2D-NMR were used to investigate structures of polyunsaturated free fatty acids [25]. A quantum chemical study of the folding of EPA and DHA was reported by Bagheri et al. [26] and Venianakis et al. [27,28], which provided low-energy structures of ω-3 fatty acids in the liquid state based on NMR and DFT calculations of ^1^H NMR chemical shifts. Emphasis has been given to an atomistic structural model of DHA.

Despite numerous conformational studies of FFAs in the liquid state, little is known about the effect of solvent polarity and hydrogen bond properties. We report herein detailed structural studies of the monounsaturated caproleic (dec-9-enoic) acid and oleic (octadec-9-enoic) acid, and the ω-3 polyunsaturated FFAs, α-linolenic acid, EPA, and DHA in chloroform and DMSO solution, with the combined use of NMR (variable concentration 1D transient NOEs and variable temperature NMR chemical shifts of the carboxylic groups) and DFT calculations. The results are compared with previous studies in the liquid state [27,28]. DFT atomistic structural models, in agreement with the NMR data, are critically evaluated.

## 2. Results and Discussion

### 2.1. ^1^H NMR Chemical Shifts of Carboxylic Protons and 1D ^1^H NMR Transient NOE in CDCl_3_: Variable Temperature and Concentration Studies

The chemical shifts of the carboxylic protons, δ(COOH), and phenol OH group, δ(OH), are very informative criteria for the investigation of various types of hydrogen bond interactions [28,29,30,31]. δ(COOH) and δ(OH) are deshielded in the presence of hydrogen bond interactions, and linear correlations between ^1^H NMR chemical shifts and hydrogen bond distances have been reported [30,31]. Temperature also has a significant effect; thus, by increasing the temperature, the ^1^H NMR chemical shifts are shielded due to the breaking of hydrogen bond interactions (negative temperature coefficients, Δδ/ΔΤ). The ^1^H NMR resonances of the COOH groups display broad signals at room temperature in CDCl_3_. The broadening is mainly due to the intermolecular proton exchange of the COOH group with the residual H_2_O in CDCl_3_ solution. The use of low concentrations (c < 100 mM) has a profound effect on the proton exchange rate, which results in excessive line broadening and variable chemical shifts. The use of activated molecular shifts in the bottom of the NMR tube, but outside the active volume of the NMR coil, resulted in a significant reduction in the line widths which allowed the accurate determination of the chemical shifts and Δδ/ΔΤ values.

**Figure 1:**
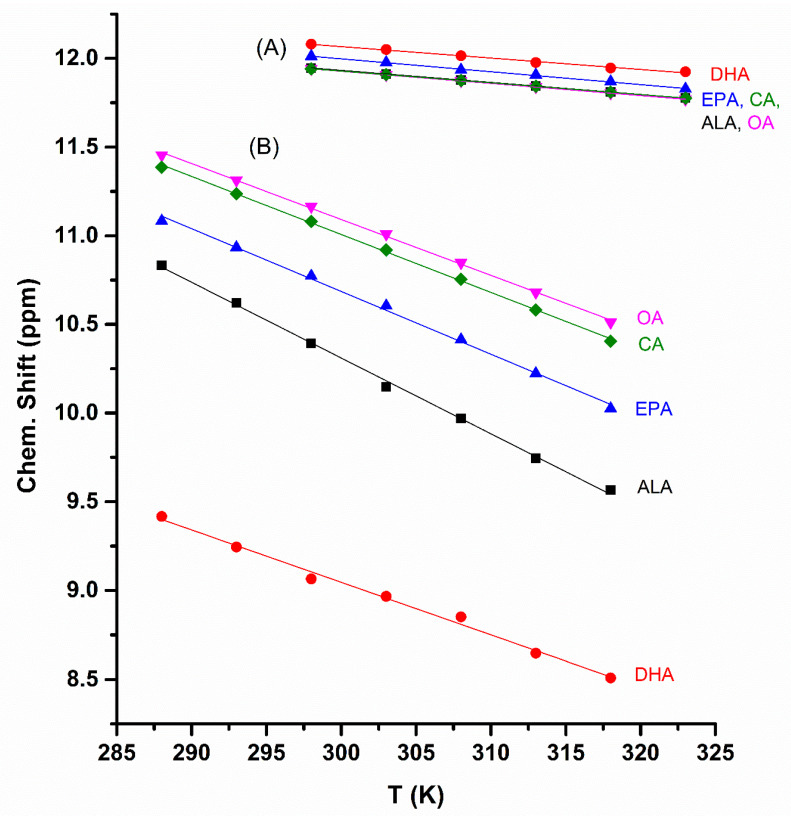
The temperature dependence of the COOH ^1^H NMR chemical shifts of caproleic acid (CA), oleic acid (OA), ALA, EPA and DHA in DMSO-d_6_, c = 20 mM (**A**) and CDCl_3_, c = 40 mM (**B**).

δ(COOH) chemical shifts at 298 K, Δδ/ΔΤ (ppb K^−1^), and statistical analysis (coefficient of linear regression R^2^ and intercept) of the data of Figure 1 are shown in Table 1. The temperature-dependent changes of the chemical shifts are linear and the derived Δδ/ΔT values, with R^2^ > 0.992, cover a range of −42.74 to −29.52 ppb K^−1^. These values are significantly larger, in absolute terms, than those obtained in the liquid state for caproleic acid, oleic acid, α-linolenic acid, EPA and DHA (−16.43 to −10.32 ppb K^−1^) [28] (Table 1) and semi-fluorinated oleic, elaidic and stearic acids [32]. This shows that by increasing the temperature, the intermolecular hydrogen bonds are more readily broken in CDCl_3_ solution than those in the liquid state.

Numerous investigations of various carboxylic acids in CCl_4_ and CHCl_3_ were interpreted in terms of mixtures of cyclic and linear dimers, cyclic and linear trimers, and monomers [33,34,35,36,37,38,39]. For long-chain carboxylic acids, such as in FFAs, the formation of centro-symmetric hydrogen bond species through carboxylic groups appears to be the major structural mode. Thus, the single crystal X-ray structural analysis of linoleic acid, α-linolenic acid and arachidonic acid [40] showed the formation of centro-symmetric cyclic hydrogen bonds, which deviate from planarity by 26.7°, with short O^…^O distances of 2.67 Å. Figure 1 and the data of Table 1 demonstrate that caproleic acid, oleic acid, ALA and EPA form intermolecular hydrogen bond interactions, since the chemical shifts of the carboxylic protons are strongly deshielded (11.17 to 10.39 ppm, at 298 K) (Table 1). The centro-symmetric hydrogen bond species through carboxylic groups, therefore, are the major components in CDCl_3_ solution. This is in agreement with literature data [41] that the monomeric species in the liquid state for octanoic, nonanoic, decanoic and undecanoic acids are only 1% to 3% in the temperature range of 280 to 360 K.

The chemical shifts of the carboxylic groups of CA, OA, ALA, and EPA in CDCl_3_ (Table 1) are slightly more shielded by 1.17 to 0.14 ppm relative to those in the liquid state [28]. This can be attributed to the major role of the centro-symmetric cyclic dimers relative to the contributions of other components of the equilibrium mixtures in both liquid state and CDCl_3_ solution. Detailed dilution studies of caproleic acid in the range of 400 to 1 mM showed a very significant shielding in the concentration range below 15 mM due to the increased contribution of the monomeric species. Thus, at 10 mM, the chemical shift of caproleic acid is ~8.6 ppm, while that of oleic acid, at 2 mM, is ~9.3 ppm. Further research is needed to determine the precise values of dimer-to-monomer dissociation constants, which apparently depend on the length of the side chain and the presence of multiple cis double bonds, as in the case of ω-3 fatty acids, which result in a significant ‘kink’ into the chain (see discussion below).

DHA is a particular case, since the chemical shift of the carboxylic group is strongly shielded (δ = 9.07 ppm at 298 K). The chemical shift at 298 K is very similar to that in the liquid state (8.60 ppm) [28], and the Δδ/ΔΤ value (−29.52 ppb K^−1^) is larger to that in the liquid state. It can, therefore, be concluded that for the dimeric DHA in CDCl_3_, a structural mode of intermolecular hydrogen bonds through carboxylic groups and an intermolecular hydrogen bond between the carboxylic group of one molecule and the terminal double bond of the second molecule of DHA plays a significant role, as in the case of the liquid state [28]. The OH^…^π hydrogen bond has been suggested to have a significant structural role in bioorganic chemistry [42,43] and biochemistry [44,45].

**Figure 2:**
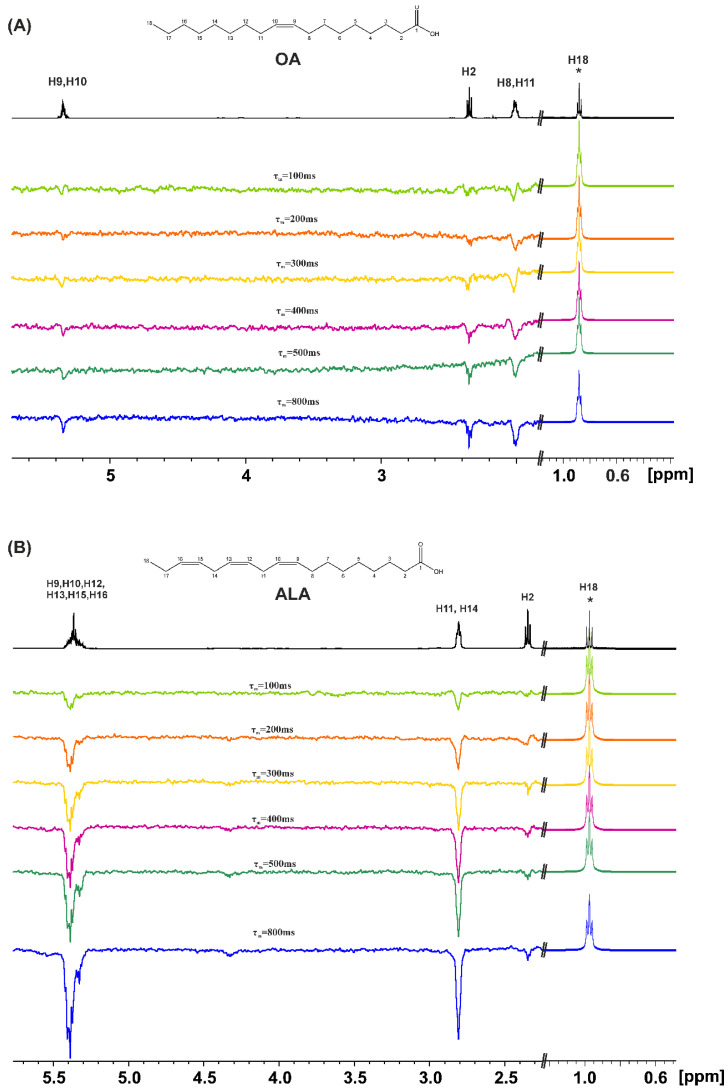
The 1D transient NOE NMR spectra of: (**A**) oleic acid (OA) and (**B**) α-linolenic acid (ALA), concentration = 20 mM in CDCl_3_ solution (number of scans = 512, T = 298 K, T_acq_ = 4.09 s, relaxation delay = 4 s), using various τ_m_ values. The amplitude of the excited CH_3_ group (denoted with the asterisk (*)) is reduced by a factor of 30 relative to the amplitude of the rest of the NOE signals.

The 1D transient NOE experiments were performed for caproleic acid (CA), oleic acid (OA), α-linolenic acid (ALA), EPA, and DHA using various concentrations (100 mM, 50 mM, and 20 mM) and mixing times, τ_m_, in CDCl_3_. Figure 2 shows the 1D NOE NMR spectra of oleic acid (OA) and α-linolenic acid (ALA) (concentration = 20 mM), using τ_m_ values in the range of 100 to 800 ms, with selective excitation of the CH_3_– group. Even for a short τ_m_ = 100 ms, there are weak NOE connectivities with the CH_2_–CH_2_–COOH protons which are antiphase with respect to the irradiated CH_3_– group. This is due to the formation of low molecular weight hydrogen-bonded species with correlation times for molecular tumbling, τ_c_, within the extreme narrowing condition (ω_ο_τ_c_ << 1) in the concentration range of 100 to 20 mM. By increasing τ_m_, an approximately linear increase in the amplitude of the NOE signal intensities is observed [33]; this shows that the NOE is due to the through space proximity of the CH_3_ group and the CH_2_–CH_2_–COOH protons in the hydrogen bond species rather than due to spin diffusion through the chain of the CH bonds.

**Figure 3:**
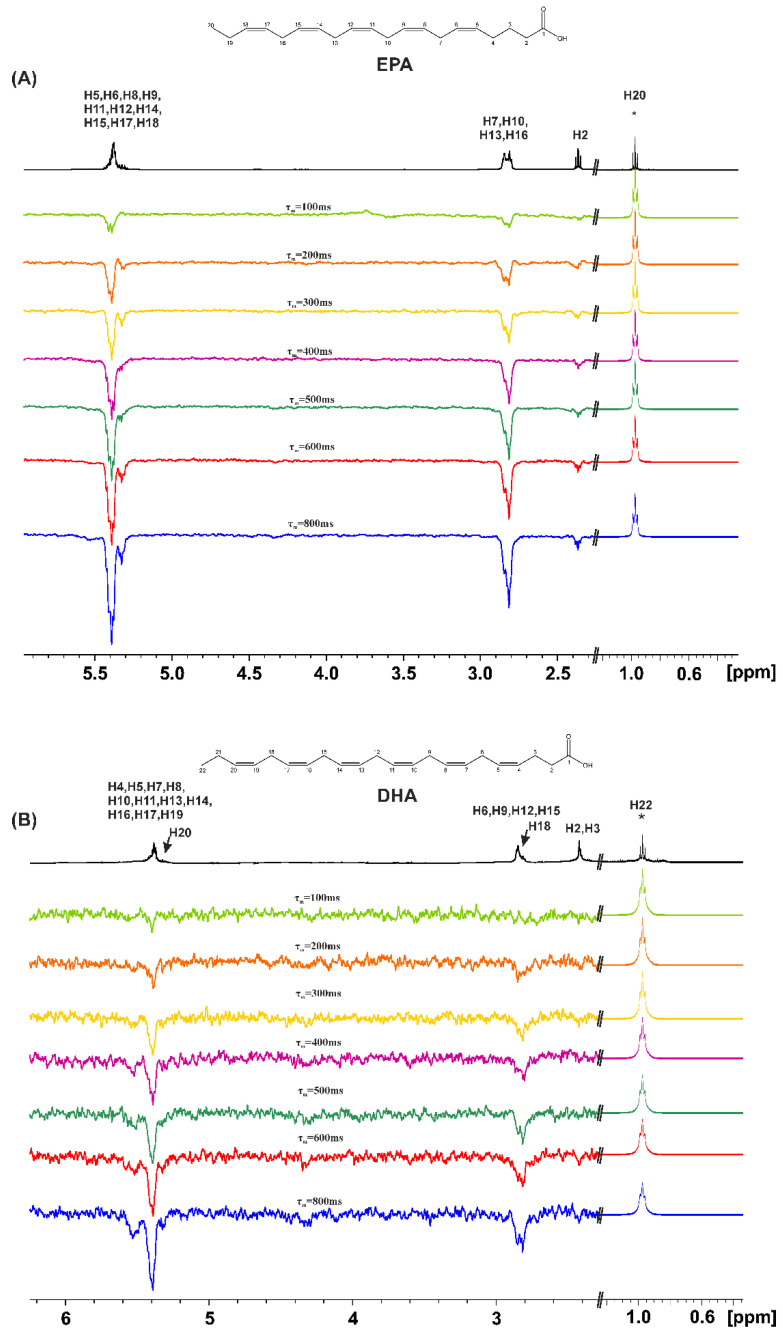
The 1D transient NOE NMR spectra of: (**A**) EPA and (**B**) DHA, concentration = 20 mM in CDCl_3_ at 298 K (number of scans = 512, T_acq_ = 4.09 s, relaxation delay = 4 s), using various τ_m_ values. The amplitude of the excited CH_3_ group (denoted with the asterisk (*)) is reduced by a factor of 30 relative to the amplitude of the rest of the NOE signals.

Similar results were obtained with EPA (Figure 3A). The magnitude of all the NOE signal intensities of DHA (Figure 3B), however, is significantly reduced relative to those of OA, ALA and EPA. This can be attributed to the formation of low molecular weight hydrogen-bonded aggregates in the range of minimum NOE signal intensities, i.e., ω_ο_τ_c_~1.

The 1D transient NOE NMR spectra of the caproleic acid (CA), with selective excitation of the α-CH_2_ protons, is shown in Figure S1A. As in the case of OA, ALA, EPA, and DHA, the NOE connectivities are anti-phase with respect to the α-CH_2_ group. The magnitude of NOEs, however, with the terminal CH(9) = CH_2_(10) protons was significantly reduced compared with those observed between α-CH_2_ and the terminal CH_3_- groups of OA, ALA and EPA. This can be attributed to the minor formation of hydrogen bond interdigitated aggregates.

### 2.2. Variable Temperature ^1^H NMR Chemical Shifts of Carboxylic Protons and 1D ^1^H NMR Transient NOE in DMSO-d_6_

Exchange broadening, due to the intermolecular proton exchange between COOH groups and residual H_2_O, is significantly reduced in DMSO-d_6_ because of its strong hydrogen bond and solvation ability. Therefore, δ(COOH) and Δδ/ΔT values can be determined accurately. The chemical shifts of the carboxylic protons, δ(COOH), in DMSO-d_6_ solution (c = 20 mM) are very similar and appear in a very narrow chemical shift range for all the FFAs (11.94–12.08 ppm) and are more deshielded relative to those in CDCl_3_ (Table 1 and Figure 1). This shows that the centro-symmetric cyclic dimers do not exist in DMSO-d_6_ due to the strong hydrogen bond and solvation ability of the DMSO molecules. In DHA, the flip–flop process between the intermolecular centro-symmetric bonds through the carboxylic groups and an intermolecular hydrogen bond between the carboxylic group of one molecule and the terminal double bond of the second molecule of DHA is also eliminated in DMSO solution. Further confirmation was also obtained from the Δδ/ΔT values in DMSO-d_6_ (−6.62 to −7.72 ppb K^−1^), which are significantly smaller, in absolute terms, than those in CDCl_3_. This demonstrates that the effect of increasing the temperature results in significantly less pronounced breaking of hydrogen bond interactions in DMSO-d_6_ relative to those in CDCl_3_ solution.

The great hydrogen bond and solvation ability of DMSO is clearly demonstrated from variable temperature experiments of an equimolar mixture of caproleic acid and DMSO-d_6_. The chemical shift of the carboxylic proton at 298 K (δ = 11.90 ppm) and its temperature coefficient (Δδ/ΔΤ = −6.77 ppb K^−1^) are identical to those obtained in dilute DMSO-d_6_ solution (c = 20 mM, Table 1). The following can be concluded in DMSO solution: (i) the centro-symmetric cyclic dimers through the carboxylic groups do not exist and (ii) the solvation state of the carboxylic group involves a single discrete molecule of DMSO which greatly facilitated the DFT calculations (see discussion below).

**Figure 4:**
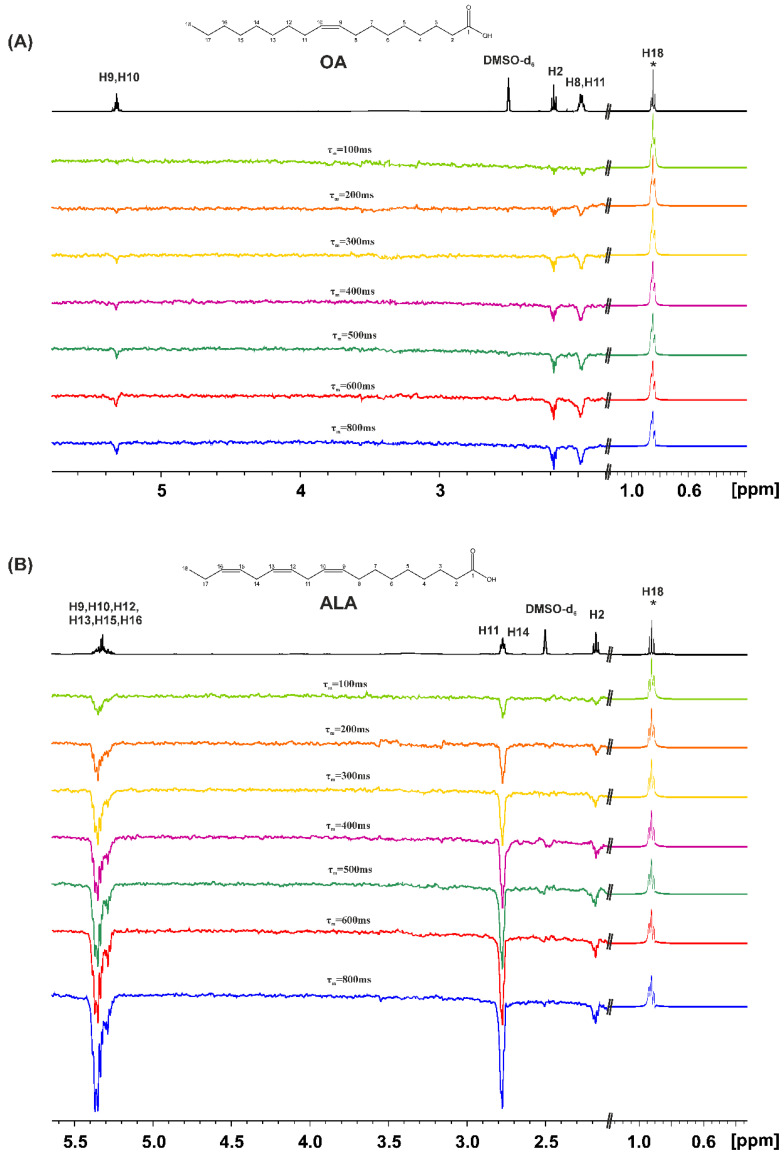
The 1D transient NOE NMR spectra of: (**A**) oleic acid (OA) and (**B**) α-linolenic acid (ALA), concentration = 20 mM in DMSO-d_6_ solution (number of scans = 512, T = 298 K, T_acq_ = 4.09 s, relaxation delay = 4 s), using various τ_m_ values. The amplitude of the excited CH_3_ group (denoted with the asterisk (*)) is reduced by a factor of 30 relative to the amplitude of the rest of the NOE signals.

The 1D transient NOE experiments were performed for the FFAs in DMSO-d_6_ with a concentration c = 20 mM. Figure 4 shows NOE NMR spectra of OA and ALA using τ_m_ values in the range of 100 to 800 ms with selective excitation of the terminal CH_3_ group. Even for τ_m_ = 100 ms, there are NOEs with the CH_2_–CH_2_–COOH protons which are antiphase with respect to the CH_3_ group. This is due to the formation of low molecular weight hydrogen-bonded aggregates with τ_c_ values within the extreme narrowing condition (ω_o_τ_m_ << 1). By increasing τ_m_, an increase in the amplitude of the NOE connectivities is observed, which can be attributed to, through space, the proximity of the CH_3_ group and the CH_2_–CH_2_–COOH protons in the hydrogen bond species rather than due to spin diffusion through the chain of the CH bonds.

Similar results were obtained with EPA and DHA (Figure S2). Selective excitation of the terminal CH_3_ group results in anti-phase NOE connectivities with H2 and H3, even for τ_m_ = 100 ms. This demonstrates the proximity, through space, of the CH_3_ group and the CH_2_–CH_2_–COOH protons in the low molecular weight hydrogen bond interdigitated aggregates within the extreme narrowing condition (ω_o_τ_m_ << 1).

The 1D transient NOE NMR spectra of caproleic acid (CA), using various τ_m_ values with the selective excitation of α-CH_2_ protons, are shown in Figure S1B. The magnitude of the anti-phase NOEs, with the terminal CH(9)=CH_2_(10) protons, was found to be significantly less than those observed between α-CH_2_ and the terminal CH_3_-groups of OA, ALA, EPA and DHA. This can be attributed to the minor formation of hydrogen bond interdigitated species.

From the above, it is evident that the parallel and antiparallel interdigitated structures of low molecular weight are an intrinsic property of the FFAs, which are not strongly affected by the length and the degree of unsaturation of the chain and the hydrogen bond ability of the solvent (chloroform vs. DMSO).

### 2.3. DFT Calculations in CHCl_3_—Comparison with the Liquid State

**Figure 5:**
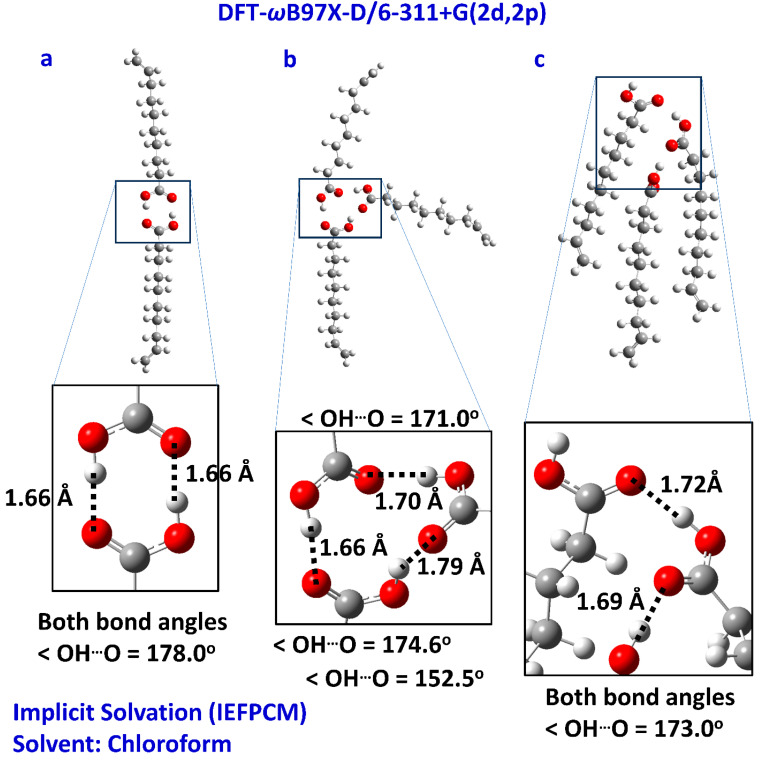
Optimized structures of caproleic acid: (**a**) dimeric structure forming OH^…^OC centro-symmetric hydrogen bonds. (**b**) Cyclic trimeric structure and (**c**) linear trimeric structure in implicit solvation (IEFPCM chloroform).

**Figure 6:**
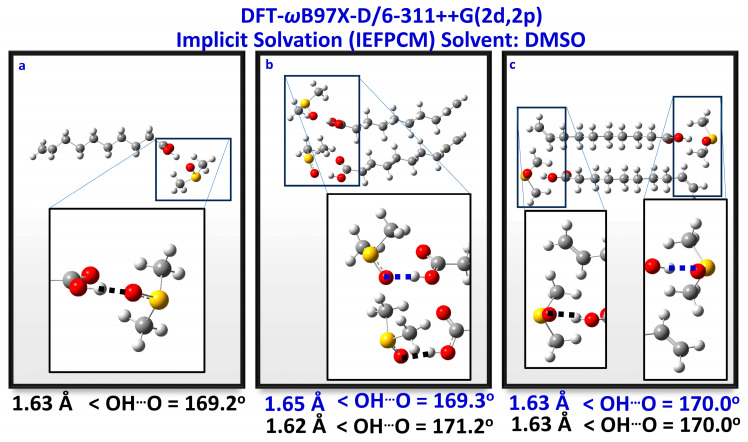
Optimized structures of caproleic acid (CA) with a discrete solvation molecule of DMSO on the carboxylic group: single molecule of CA (**a**); dimeric structures of CA in parallel (**b**) and antiparallel configuration (**c**).

Computational approaches have been proven to be very successful in elucidating the structural and spectroscopic experimental data of free fatty acids in the liquid state [27,28]. Moreover, this approach has been used as a predictive tool in biotechnology for predesigned properties of functional free fatty acid aggregates by tuning their interatomic interactions in organic materials [46]. Based on the state of the FFA carboxylic proton, it was concluded that the FFA in the proper solution can be used as a transport or catalytic medium [47]. The present computations were designed to investigate possible inter- and intramolecular interactions that justify the experimental δ(COOH) and 1D NOE NMR results presented above. Caproleic acid was investigated in the dimeric structure forming O–H^…^O=C centro-symmetric hydrogen bonds (Figure 5a) in the cyclic trimeric (Figure 5b) and linear trimeric (Figure 5c) structures in implicit solvation (IEFPCM-chloroform). In the centro-symmetric dimeric structure (Figure 5a), the dihedral angle defined by the four oxygen atoms of the carboxylic groups is only 0.8°, the (O)H^…^O(C) and O^…^O hydrogen bond distances are 1.66 and 2.65 Å, respectively, and the O–H^…^O bond angle indicates a nearly linear (178.0°) hydrogen bond interaction. These values can be compared with the O^…^O distance of 2.67 Å and deviation from planarity of 26.7° in the single-crystal X-ray structure of linolenic acid, α-linolenic acid and arachidonic acid [40]. The experimental chemical shifts of caproleic acid (δ = 11.08 ppm at 298 K, Table 1) are rather indistinguishable on the basis of the structures of Figure 6a,b (13.6 ppm and 12.9/11.2/10/7 ppm, respectively, Table 2). In the linear aggregate structure shown in Figure 6c, the presence of a carboxylic group which does not participate in hydrogen bond interactions (12.2/12.2/6.8 ppm) results in an average chemical shift of 10.4 ppm. A minor contribution of the structural model Figure 6c, therefore, could account for the deviation of the experimental data from the computational data of the structures Figure 5a,b. Moreover, the hydrophobic effect of the carbon chains in Figure 6c seems to play an antagonistic role with respect to the cyclic structure shown in Figure 5b.

Computations were also performed with the tetrameric caproleic acid in a parallel orientation similar to the single crystal X-ray structures of free fatty acids [40] and in an antiparallel orientation in agreement with the experimental weak NOE data of the through-space proximity of the α-CH_2_ and the terminal CH(9)=CH_2_(10) olefinic protons. A similar methodology was used for the interpretation of the NOEs observed in the liquid state for CA, OA, ALA, and EPA [28]. The calculated chemical shifts of the carboxylic proton for the tetrameric CA, in the parallel configuration, vary between 14.3 and 13.0 ppm, while in the antiparallel configuration, they vary between 13.8 and 13.2 ppm. The chemical shift difference of 1.3 ppm observed for the parallel arrangement can be attributed to the two interacting cyclic hydrogen bonds.

### 2.4. DFT Calculations in DMSO

The DFT calculated ^1^H NMR chemical shifts of the carboxylic protons with a discrete solvation molecule of DMSO were investigated in the case of a single molecule of CA and a CA dimer with parallel and antiparallel arrangements (Figure 6 and Table 3). The representative system is a molecule of caproleic acid interacting with a single discrete DMSO molecule, explicitly present in the design, while the DMSO solvent is present implicitly (Figure 6a). To this interacting pair, another one was added and oriented in parallel and antiparallel arrangements (Figure 6b,c). These configurations were chosen to explore possible interactions between DMSO and the proton of the carboxylic group or the double bond of the caproleic acid and the proton of the carboxylic group. The results presented in Table 3 indicate that the orientations of Figure 6 produce practically indistinguishable δ(COOH) chemical shifts with values ranging from 13.4 to 14.2 ppm. In all cases, very strong hydrogen bond interactions of the carboxylic protons with the oxygen of the DMSO molecule were observed with OH^…^O distances of 1.59 to 1.63 Å and bond angles of 169.2° to 171.2°. These hydrogen bond distances are significantly shorter than those observed in the centro-symmetric hydrogen bond interactions through the carboxylic groups with OH^…^O distances of 1.66 Å.

The results of the complexation energy of the caproleic dimer in Figure 5a and the caproleic acid–DMSO complex in Figure 6a are very informative (Table 2 and Table 3). For the structure shown in Figure 5a, the complexation energy is −21.2 kcal/mole, while it is −18.0 kcal/mole for the 6a (DFT-ωB97X-D/aug-cc-pVDZ, in the gas phase). Given that the centro-symmetric hydrogen bond is double while in the caproleic-DMSO complex only one hydrogen bond is formed, DMSO seems to be the most potent antagonist for this interaction. Additionally, the relative electronic energy for the cyclic trimer in chloroform (present implicitly) is 0.0 kcal/mole, while for the linear trimer in DMSO, it is −8.9 kcal/mole. The antiparallel configuration for the CA-DMSO dimer is more stable by 2.7 kcal/mole.

Similar results were obtained with oleic acid, α-linolenic acid, EPA, and DHA. The OH^…^O hydrogen bond distances (1.64 to 1.63 Å), the O–H^…^O hydrogen bond angles (168.5° to 169.1°) and the COOH chemical shifts (δ = 13.1 to 13.5 ppm) are indicative of a very strong intermolecular hydrogen bond with a single solvation molecule of DMSO (Table 3 and Figure S3).

## 3. Materials and Methods

### 3.1. Chemicals and Reagents

Caproleic acid, purity ≥ 96%, oleic acid, purity ≥ 99% (GC), and α-linolenic acid, purity ≥ 99%, were purchased from Sigma-Aldrich, Chemie, GmbH, Taufkirchen, Germany. EPA, purity > 99%, and DHA, purity > 99%, were purchased from Larodan AB, Karolinska Institutet, Solna, Sweden. Chloroform-d_1_ and DMSO-d_6_, 99.8%, were obtained from Deutero, GmbH, Kastellaun, Germany. Molecular sieves (3Å) were obtained from Sigma-Aldrich and activation was achieved by heating at 200–230 °C for 24 h and the use of a high vacuum for 3 h.

### 3.2. ^1^H NMR Chemical Shifts and 1D ^1^H NMR Transient NOE: Variable Temperature and Concentration Studies

Variable temperature ^1^H NMR experiments were performed on a Bruker AVANCE NEO 500 spectrometer controlled by the software TopSpin 3.2. The temperature was maintained and measured with an accuracy of ±0.1 °C. Chemical shifts were reported with respect to the solvent residual signal (CDCl_3_/DMSO-d_6_). The correction of temperature dependencies of the chemical shifts of the solvents was not applied, since they are very small [48,49], in absolute terms, falling well below the anticipated range of Δδ/ΔT values of the carboxylic protons. A variable concentration (100 to 20 mM) 1D transient NOE experiments [50,51,52] was performed with the use of the pulse program selnogp with pulse field gradients (PFGs). The recovery delay was set to 200 μs, and the shaped pulse was set to 50 ms [28]. NMR experiments were performed on freshly prepared solutions to avoid the formation of significant amounts of primary and secondary oxidation products [53,54].

### 3.3. DFT Calculations of ^1^H NMR Chemical Shifts and Complexation Energies

All geometries were optimized at the DFT-*ω*B97X-D level of theory [55,56]. The selected functional performs very well for hydrogen-bonded complexes [57]. Three basis sets were adopted (aug-cc-pVDZ, 6-311++G(2d,2p), and 6-31+G(d,p)) and adjusted at the relative molecular system size and computational cost. The selected functional is a range-separated functional, based on a modified Becke’s 97 functional with added dispersion corrections. It comprises a 22% Hartree–Fock exchange for the short range and 100% Hartree–Fock for the long range. A standard error function with a default range separation parameter value of ω = 0.2 was applied for the intermediate region. Tight optimization criteria were employed (RMS force = 1 × 10^−5^), while subsequent frequency calculations located no imaginary frequencies, confirming that the optimized structures are true minima. The GIAO (Gauge-Independent Atomic Orbital) [58] was employed to calculate the NMR spectrum. The counterpoise corrections included the basis set superposition error (BSSE) in the complexation energy calculations [59]. The Polarizable Continuum Model (PCM) with the integral equation formalism variant (IEFPCM) was employed for implicit solvation [60]. The computations were run on the FASRC Odyssey cluster supported by the FAS Division of Science Research Computing Group at Harvard University.

## 4. Conclusions

The combined use of variable temperature and concentration ^1^H NMR chemical shifts of the carboxylic protons, variable concentration transient 1D NOE experiments, and DFT calculations of ^1^H NMR chemical shifts are an effective approach to investigate a variety of low-energy structures of unsaturated and polyunsaturated FFAs in chloroform and DMSO solution. More specifically:

(a) Caproleic acid, oleic acid, α-linolenic acid, and EPA, in various concentrations in chloroform solution (c = 100 to 20 mM), exist mainly in the form of hydrogen-bonded dimers through carboxylic groups in an equilibrium of parallel and antiparallel interdigitated structures. The correlation times for molecular tumbling are within the extreme narrowing condition for all FFAs; therefore, the hydrogen-bonded aggregates are of low molecular weight. In DHA, a structural model of an intermolecular hydrogen bond through carboxylic groups and an intermolecular hydrogen bond between the carboxylic group of one molecule and the terminal double bond of a second molecule is shown to play a role, as in the case of the liquid state [28].
(b) In DMSO solution, at low concentration (c = 20 mM), all the FFAs investigated show a strong hydrogen bond interaction of a single discrete solvation molecule of DMSO with the carboxylic group, without hydrogen-bonded dimers through the carboxylic groups. The 1D NOE experiments and DFT calculations show the presence of parallel and antiparallel interdigitated configurations of low molecular weight within the extreme narrowing condition (ω_ο_τ_c_ << 1).
(c) The parallel and antiparallel interdigitated structures of low molecular weight are an intrinsic property of the FFAs, which are not strongly affected by the length and the degree of unsaturation of the chain and the hydrogen bond ability of the solvent.

The present study shows the great conformational flexibility of mono- and polyunsaturated FFAs in various solvents and the importance of the combined use of NMR and DFT studies [18,19,27,28,61,62,63,64]. The significant conformational flexibility of FFAs was also considered to be the main reason that their location in the binding site FA7 in the human serum albumin could not be determined accurately [18,19,64] in the available X-ray structural data [65,66,67]. The structures of free fatty acids and their oxidation products [53,54], in various solvents with varying hydrogen bond and solvation abilities, are currently under investigation with the combined use of NMR and DFT studies.

## Figures and Tables

**Table 1:** δ(COOH) chemical shifts at 298 K, Δδ/ΔΤ, and statistical analysis (R^2^ and intercept) of the data of Figure 1 of δ(^1^H) vs. T(K) of the free fatty acids in CDCl_3_ (c = 40 mM), DMSO-d_6_ (c = 20 mM) and in the liquid state.

	CDCl_3_				DMSO-d_6_				Liquid State ^a^			
FFA	δ (ppm)	R^2^	Δδ/ΔΤ (ppb K^−1^)	Inter.	δ (ppm)	R^2^	Δδ/ΔΤ (ppb K^−1^)	Inter.	δ (ppm)	R^2^	Δδ/ΔΤ (ppb K^−1^)	Inter.
CA	11.08	0.999	−32.69	20.81	11.94	0.999	−6.62	13.92	12.25	0.999	−11.31	15.98
OA	11.17	0.999	−31.50	20.54	11.94	0.999	−6.88	13.99	12.13	0.998	−10.32	15.21
ALA	10.39	0.998	−42.74	23.13	11.95	0.999	−6.79	13.97	10.88	0.998	−13.06	14.76
EPA	10.77	0.997	−35.41	21.31	12.01	0.997	−7.27	14.18	10.91	0.999	−14.38	14.19
DHA	9.07	0.992	−29.52	17.90	12.08	0.993	−6.45	14.00	8.60	0.986	−16.43	13.51

^a^ Ref. [28].

**Table 2:** Calculated δ(COOH) chemical shifts of the free fatty acids under study in implicit solvation (IEFPCM-chloroform).

FFA	Intermolecular Interaction	δ(COOH) (ppm)	Complexation Energy (kcal/mole—Gas Phase)
CA dimer	COO-H^…^O=COH	13.6	−21.2 ^a^/−20.7 ^b^
CA cyclic trimer	COO-H^…^O=COH	12.9/11.2/10.7	−17.4
CA linear trimer	COO-H^…^O=COH COOH (free)	12.2/12.2 6.8	−29.2
CA tetramer parallel	COO-H^…^O=COH	14.3, 14.0, 13.7, 13.0	
CA tetramer antiparallel	COO-H^…^O=COH	13.8, 13.8, 13.8, 13.2	

^a^ *ω*B97X-D/aug-cc-pVDZ; ^b^ *ω*B97X-D/6-311+G(2d,2p).

**Table 3:** Calculated δ(COOH) chemical shifts of the free fatty acids under study with a discrete solvation molecule of DMSO.

FFA	Intermolecular Interaction	δ(COOH) (ppm)	Complexation Energy (kcal/mole—Gas Phase)
CA	COO-H^…^DMSO	13.4	−18.0 ^a^
CA dimer parallel	COO-H^…^ DMSO	14.4, 13.9 ^b^	−15.7 ^c,d^
CA dimer antiparallel	COO-H^…^ DMSO	14.1, 14.2 ^b^	−15.9 ^c,d^
OA	COO-H^…^DMSO	13.4	
ALA	COO-H^…^ DMSO	13.4	
EPA	COO-H^…^DMSO	13.1	
DHA	COO-H^…^DMSO	13.5	

^a^ ωB97X-D/aug-cc-pVDZ; ^b^ ωB97X-D /6-31+G(d,p); ^c^ ωB97X-D /6-311++G(2d,2p); ^d^ the complexation energy was calculated between the two DMSO–CA aggregates and not between a molecule of CA and a DMSO molecule.

## Data Availability

Data will be made available on request.

## Supplementary Materials

The following supporting information can be downloaded at: https://www.mdpi.com/article/10.3390/molecules28166144/s1, Figure S1: The 1D transient NOE (500 MHz) NMR spectra of caproleic acid (CA), c = 20 mM in CDCl_3_ solution (A) and c = 20 mM in DMSO-d_6_ solution (B) (number of scans = 512, T = 298 K, T_acq_ = 4.09 s, relaxation delay = 4 s) using various mixing times (τ_m_). The excited α-CH_2_ group (denoted with the asterisk (*)) is reduced by a factor of 30 relative to the amplitude of the NOE signals in the region up to 5.9 ppm. Figure S2: The 1D transient NOE NMR spectra of: (A) EPA and (B) DHA, concentration = 20 mM in DMSO-d_6_ at 298 K (number of scans = 512, T_acq_ = 4.09 s, relaxation delay = 4 s), using various τ_m_ values. The amplitude of the excited CH_3_ group (denoted with the asterisk (*)) is reduced by a factor of 30 relative to the amplitude of the rest of the NOE signals. Figure S3: Optimized structures of oleic acid (OA), α-linolenic acid (ALA), EPA and DHA with a discrete solvation molecule of DMSO on the carboxylic group.

## Sample Availability

Samples of the compounds are available from the authors.
