# Supplementary material for: Structural Studies of Monounsaturated and ω-3 Polyunsaturated Free Fatty Acids in Solution with the Combined Use οf NMR and DFT Calculations—Comparison with the Liquid State
Source: Molecules. 2023 Aug 20;28(16):6144. doi: 10.3390/molecules28166144 (PMC10459368; doi:10.3390/molecules28166144)
Supplement: Supplementary file 1 [file molecules-28-06144-s001.zip › molecules-2546710-supplementary.pdf]

Appendix A. Supplementary Material

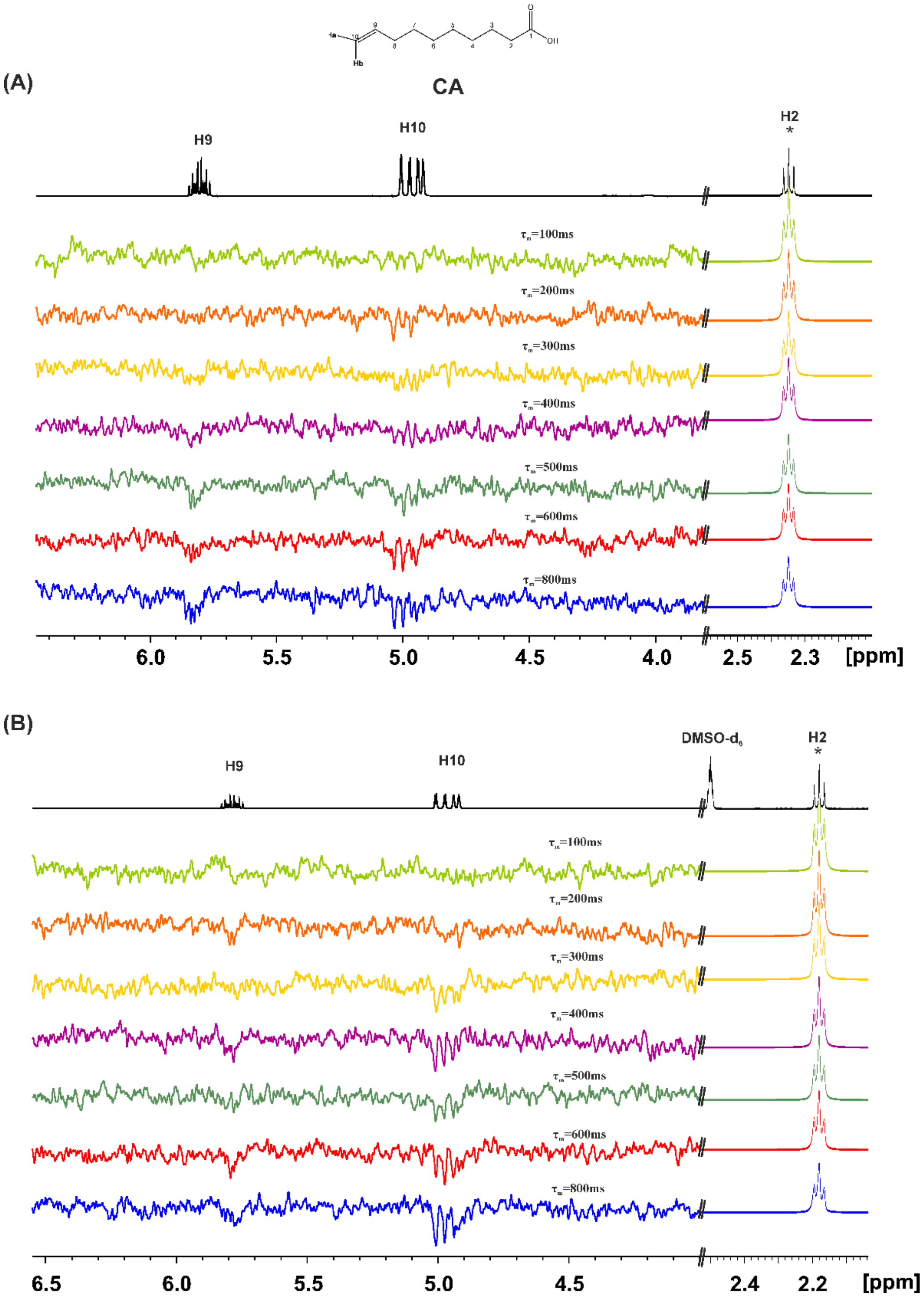

Figure S1. 1D transient NOE (500 MHz) NMR spectra of caproleic acid (CA), c = 20 mM in $CDCl_3$ solution (A) and c = 20 mM in DMSO-d_6 solution (B) (number of scans=512, T=298K, T_{acq}=4.09s, relaxation delay=4s) using various mixing times (τ_m). The excited α-CH_2 group (denoted with the asterisk (*)), is reduced by a factor of 30, relative to the amplitude of the NOE signals in the region up to 5.9 ppm.

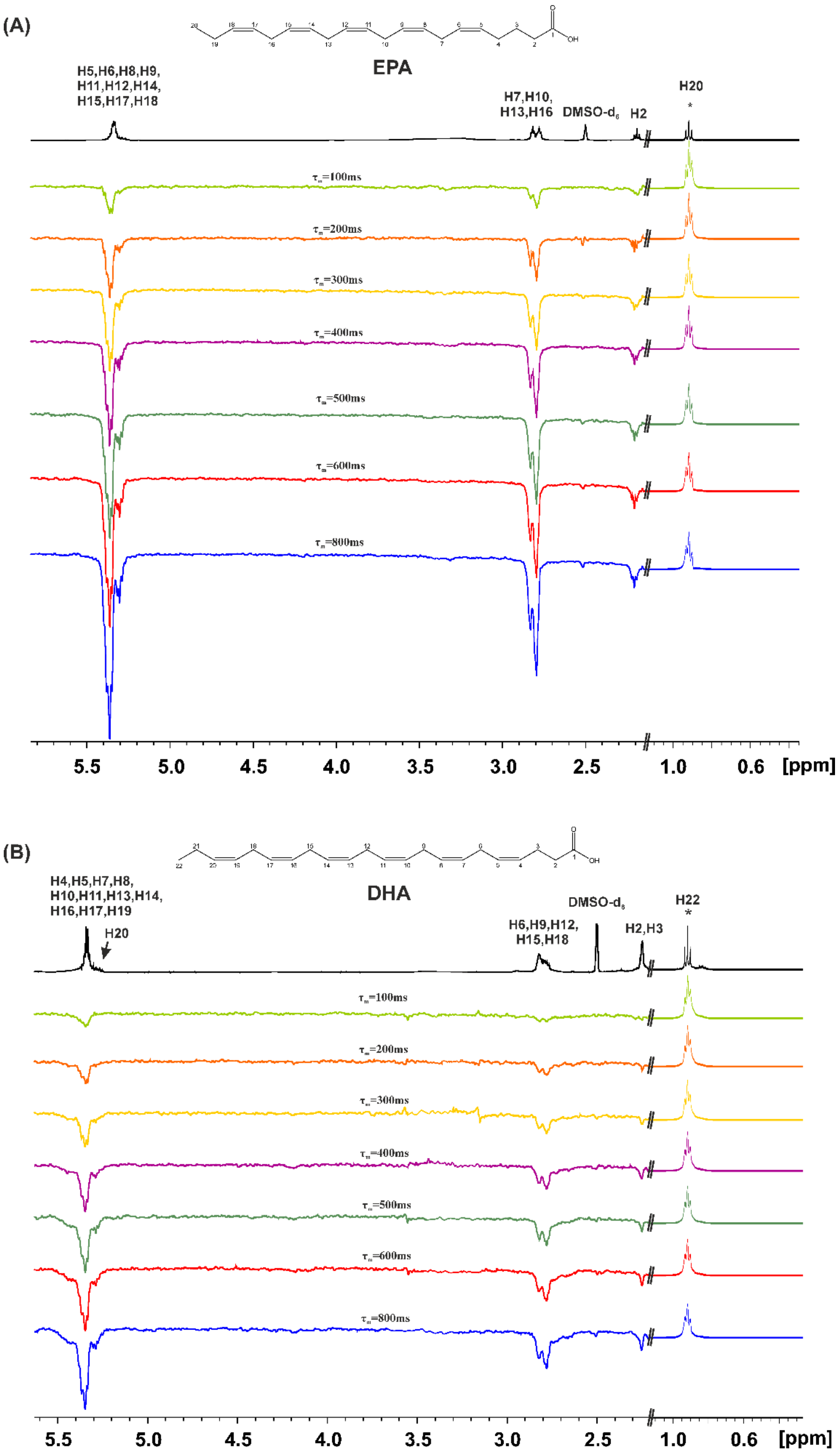

Figure S2. 1D transient NOE NMR spectra of: (A) EPA and (B) DHA, concentration = 20 mM in DMSO-d6 at 298 K (number of scans = 512, T_{acq}=4.09s, relaxation delay=4s), using various τ_m values. The amplitude of the excited CH_3– group (denoted with the asterisk (*)), is reduced by a factor of 30, relative to the amplitude of the rest of the NOE signals.

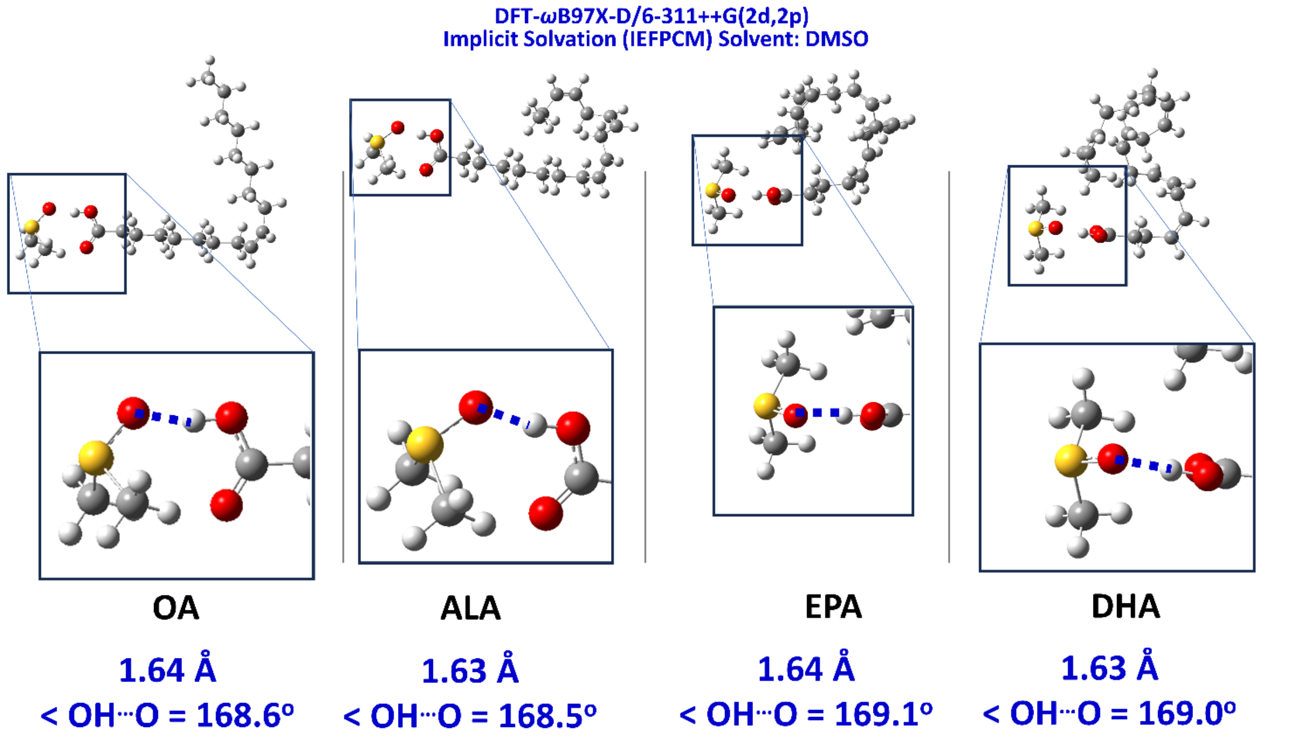

Figure S3. Optimized structures of oleic acid (OA), α-linolenic acid (ALA), EPA and DHA with a discrete solvation molecule of DMSO on the carboxylic group.
